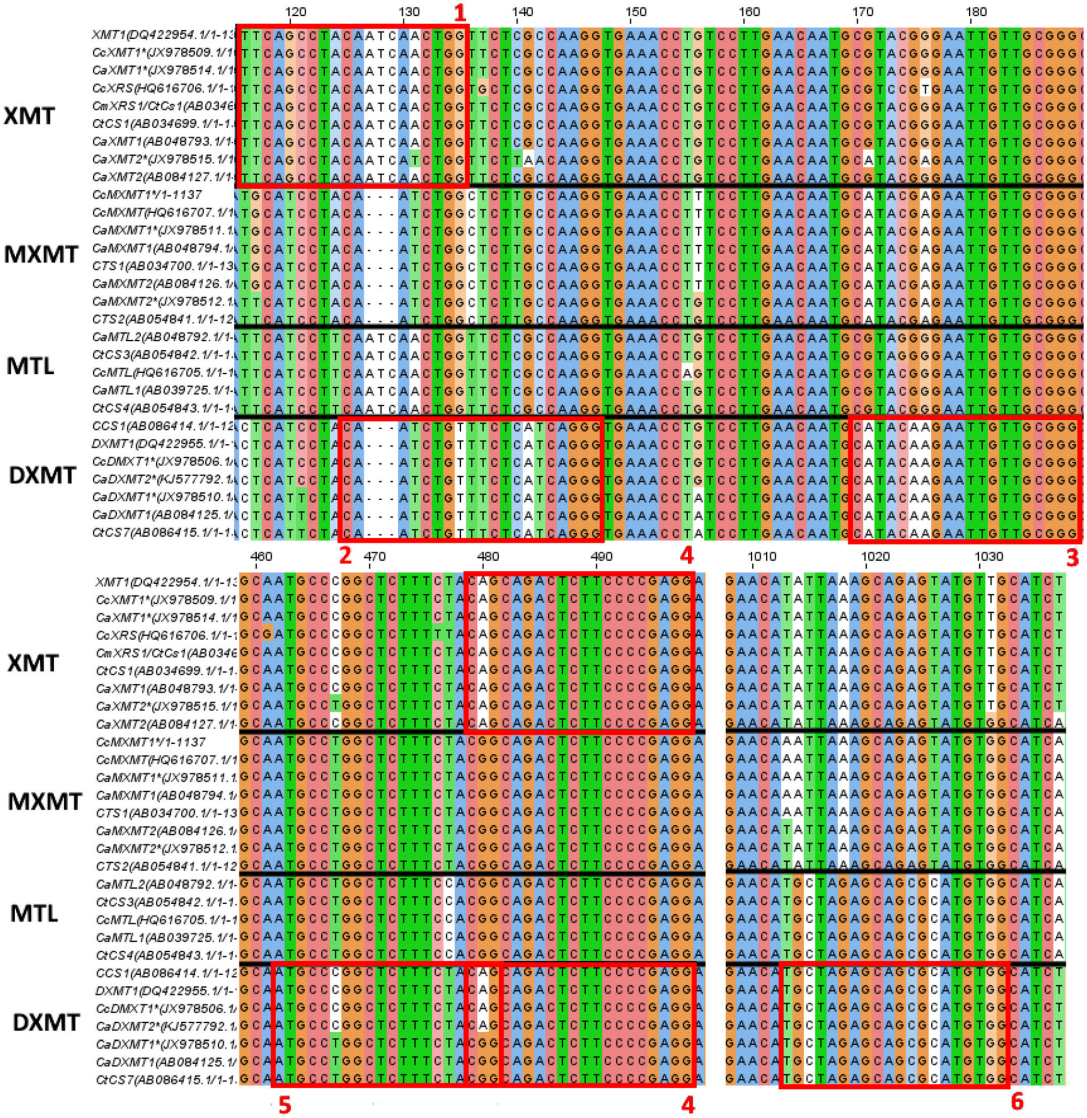# The biological feasibility and social context of gene-edited, caffeine-free coffee

**DOI:** 10.1007/s10068-022-01082-3

**Published:** 2022-05-20

**Authors:** Nils V. Leibrock, Joris Santegoets, Paul J. W. Mooijman, Filemon Yusuf, Xander C. L. Zuijdgeest, Esmée A. Zutt, Josette G. M. Jacobs, Jan G. Schaart

**Affiliations:** 1 Programme Molecular Life Sciences, Wageningen University and Research, Grumbachtalweg 129, 66121 Saarbrücken, Germany; 2 Programme Plant Sciences, Wageningen University and Research, Wageningen, The Netherlands; 3 Programme Plant Biotechnology, Wageningen University and Research, Wageningen, The Netherlands; 4 Programme Biology, Wageningen University and Research, Wageningen, The Netherlands; 5 Philosophy, Wageningen University and Research, Wageningen, The Netherlands; 6 Plant Breeding, Wageningen University and Research, Wageningen, The Netherlands

**Keywords:** CRISPR-Cas, *Coffea canephora*, *Coffea arabica*, Genetic modification, XMT, DXMT, MXMT, Caffeine pathway

## Abstract

Coffee, especially the species *Coffea arabica* and *Coffea canephora*, is one of the world’s most consumed beverages. The consumer demand for caffeine-free coffee is currently being met through chemical decaffeination processes. However, this method leads to loss of beverage quality. In this review, the feasibility of using gene editing to produce caffeine-free coffee plants is reviewed. The genes XMT (7-methylxanthosine methyltransferase) and DXMT (3,7-dimethylxanthine methyltransferase) were identified as candidate target genes for knocking out caffeine production in coffee plants. The possible effect of the knock-out of the candidate genes was assessed. Using *Agrobacterium tumefaciens*-mediated introduction of the CRISPR-Cas system to Knock out XMT or DXMT would lead to blocking caffeine biosynthesis. The use of CRISPR-Cas to genetically edit consumer products is not yet widely accepted, which may lead to societal hurdles for introducing gene-edited caffeine-free coffee cultivars onto the market. However, increased acceptance of CRISPR-Cas/gene editing on products with a clear benefit for consumers offers better prospects for gene editing efforts for caffeine-free coffee.

## Introduction

Coffee is one of the world’s most widely consumed beverages, with a global yearly consumption of over 500 billion cups (Clarke and Vitzum, 2001). The *Coffea* genus has over 100 species, but *Coffea arabica* and *Coffea canephora* are the major species for human consumption. Most coffee plants produce caffeine (1,3,7-trimethylxanthine), which acts as a natural pesticide (Nathanson, 1984). In humans, caffeine causes a psychoactive response including increased alertness and attention (Tofalo et al., 2015), but can also have a negative effect on sleep quality. Therefore, customers want caffeine-free coffee. Such a caffeine-free alternative to traditional coffee is decaffeinated coffee. Currently, caffeine-free coffee is produced by decaffeinating green coffee beans prior to roasting. Decaffeination is achieved using one of the following three processes: solvent decaffeination, water extraction or CO_2_ decaffeination. Each of these processes is based on the concept of using a solvent to extract the caffeine from the green coffee bean (Franca, 2015). However, all these techniques also remove other aromatic compounds, thus resulting in a loss of aromatic coffee quality. Moreover, the extra expenses involved in the decaffeinating process reduce overall profit. An alternative method for generating caffeine-free coffee could benefit the industry by lowering production costs and preventing a loss of taste. Such a method might be the development of a gene-edited coffee variety that produces caffeine-free coffee beans.

In the last decade, the genome of *C. canephora,* a diploid species and *C. arabica,* an allotetraploid derived from a hybridization of *C. canephora* and *C. eugenioides* (Clarindo & Carvalho, 2008; Lashermes et al., 1999), have been sequenced (Denoeud et al., 2014; Medrano et al., 2017). Whole genome sequences of *C. canephora* and *C. arabica* enable the selection of candidate gene sequences for the directed gene editing targeting of the caffeine biosynthetic pathway. To deliver the gene editing reagents into the plant, a genetic modification step is required. Advances in the genetic modification of coffee may lead to gene-edited caffeine-free coffee plants. Genetic differences between *C. arabica* and *C. canephora* may affect the success of genetic modification.

This review investigates the technical feasibility of creating gene-edited caffeine-free coffee plants. This technical feasibility is studied in two research questions:

- How can the caffeine biosynthesis pathway be modified to produce caffeine-free coffee plants?
- What is the state of the art in genetic modification of *C. arabica* and *C. canephora*?

To provide an answer to these questions, first the caffeine biosynthesis pathway, including the genes involved and possible target genes for producing a genetically modified caffeine-free coffee plant, were studied. Next, the state of the art in genetically modifying the coffee genome was investigated. Both research questions involved a systematic literature review.

## Methodology

Literature was searched and retrieved from CAB Abstracts (https://www.cabdirect.org), Scopus (https://www.scopus.com) and Web of Science (https://www.webofknowledge.com). Separate search queries were made for each research question. Because no professional translator was available during the writing of this review, literature published in any language other than English was excluded. The number of retracted articles as well as the selected inclusion criteria are described below. In total, we retracted 62 references for this systematic literature review.

The search query for the ‘caffeine biosynthesis pathway’ research question was: (“caffeine biosynthesis” OR “caffeine synth*” OR “caffeine pathway” OR “caffeine production” OR “caffeine metabolism”) AND (Coffe* OR “C* Arabica” OR “C* canephora”) NOT (health OR consumption). 315 articles were retrieved using this search query, including overlapping hits between databases. After reviewing the title & abstract and removing overlapping hits, reviews/book chapters and non-retrievable articles, 20 articles remained in the final selection (Appendix 1). Gene and protein sequences retrieved from articles in the final selection were compared using a Multiple Sequence Alignment (MSA). The MSA was created using the Clustal Omega web application version 1.2.4 of EMBL-EBI (https://www.ebi.ac.uk/Tools/msa/clustalo/) with default settings. The figures were made using Jalview 2.11.1. 2 (Waterhouse et al., 2009) with sequence harmony and multi-relief (Brandt et al., 2010) and ClustalX colouring.

The search query for the ‘genetic modification of *C. arabica* and *C. canephora*’ research question was: (Coffea OR arabica OR “Coffea Arabica” OR canephora OR “Coffea canephora”) AND (CRISPR* OR “genetic modification” OR “genetic transformation” OR “gen*e editing” OR RNAi OR transgenic OR “gene silencing” OR “genetic engineering” OR knock*out OR knock*down). A total of 309 articles was retrieved from the three databases, including overlapping hits. To ensure the most recent findings of genetic modification in coffee, only those articles published since 2010 were selected. The title and abstract of each retrieved article were reviewed by (at least) two co-authors for initial relevance. Reviews, book chapters and non-retrievable articles were not incorporated into the final selection. The final selection consisted of 16 articles (Appendix 1).

## Results

The literature retrieved is analysed for both research questions. First, the caffeine biosynthesis pathway is described and the genes involved are evaluated as candidate target genes for knock-out. Then the state of the art in genetic modification and gene editing of coffee plants is investigated.

### Modifying the caffeine biosynthesis pathway

In this section the main caffeine biosynthesis pathway in coffee plants is described, potential minor pathways are identified and the function of the enzymes involved is reviewed. This knowledge is subsequently used to identify potential candidate target genes to disrupt the biosynthesis of caffeine using gene editing.

#### Caffeine biosynthesis pathway

**Fig. 1:**
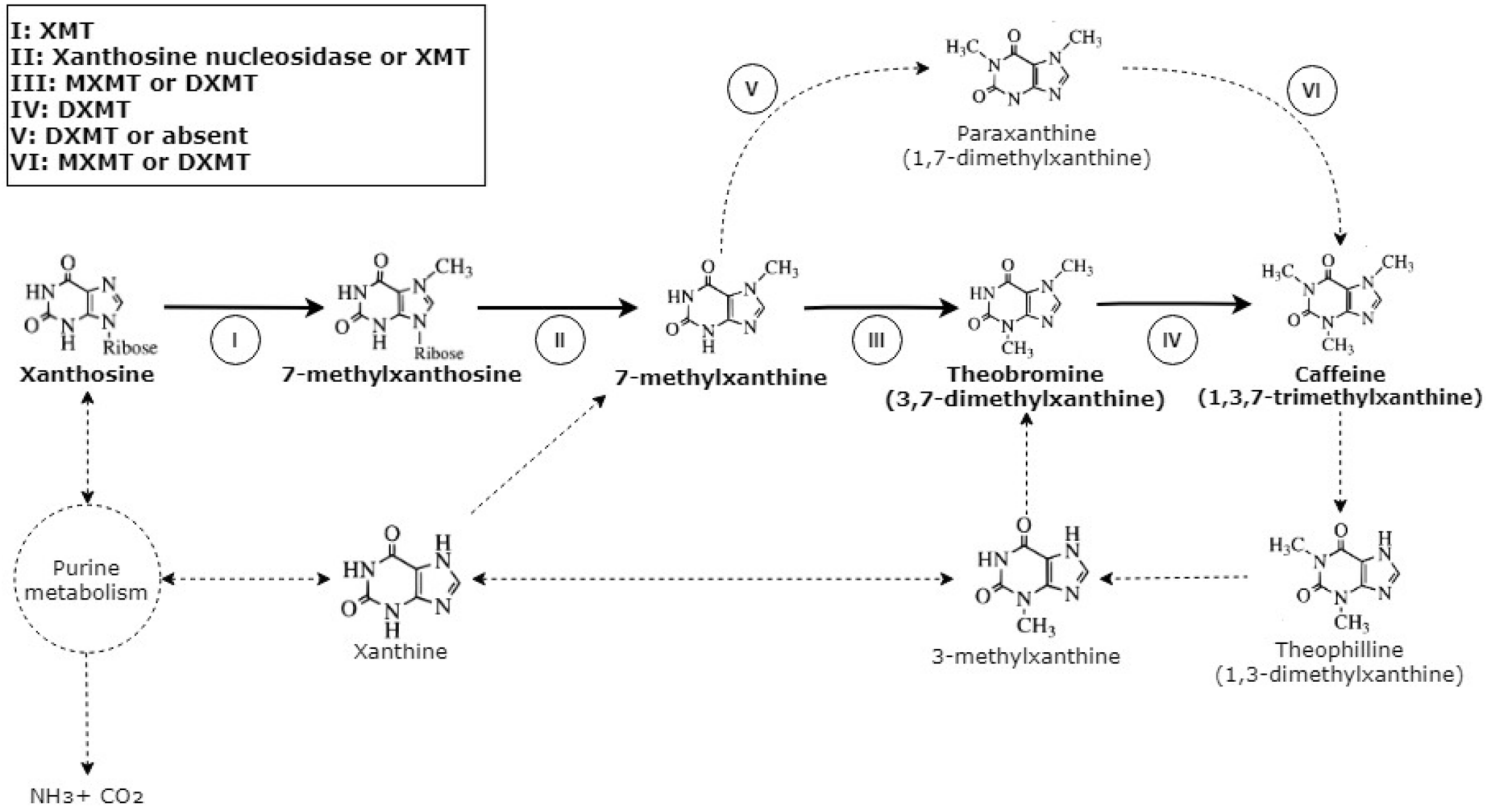
Biosynthesis (and catabolic) pathway of caffeine in coffee plants and enzymes involved. Conversion steps in the main and paraxanthine pathways are indicated by Roman numerals and the (proposed) enzymes involved are shown. The main pathway is indicated in bold. Dashed arrows illustrate minor or catabolic pathways. Note that not all conversions can occur in every species of *Coffea* as certain enzymes may not be present. Some conversions may be reversible, but the enzymes required were not described in the literature. *XMT*  xanthosine 7-methyltransferase, *MXMT*  7-methylxanthine methyltransferase, *DXMT* 3,7-dimethylxanthine methyltransferase

The main caffeine biosynthesis pathway consists of three consecutive N-methylations of xanthine derivatives, beginning with xanthosine (Fig. 1). This pathway was first described before the start of the millennium (Ashihara et al., 1996a). Individual steps and the enzymes involved have been studied extensively ever since. Figure 1 illustrates the entire caffeine biosynthesis pathway and all (proposed) enzymes involved. The precursor compounds involved are agreed upon, yet the number of enzymes involved in the pathway is up for discussion. Generally, the biosynthesis pathway of caffeine is as follows: Xanthosine (from purine metabolism) is methylated at the 7-N position by 7-methylxanthosine methyltransferase (XMT), yielding 7-methylxanthosine (step I, Fig. 1). 7-Methylxanthine is then created by removing the ribose group from 7-methylxanhosine by xanthosine nucleosidase (step II, Fig. 1). Involvement of XMT in this conversion was proposed, allowing for the direct conversion from xanthosine to 7-methylxanthine (McCarthy and McCarthy, 2007). Methylation of the 3-N position of 7-methylxanthine (step III, Fig. 1) yields theobromine (3,7-dimethylxanthine), the direct precursor of caffeine. This step is catalysed by theobromine synthase (7-methylxanthine methyltransferase; MXMT). Finally (step IV, Fig. 1), caffeine (1,3,7-trimethylxanthine) is created by the methylation of the 1-N position of theobromine catalysed by caffeine synthase (3,7-dimethylxanthine methyltransferase; DXMT). Dual functionality of DXMT was shown in vitro where DXMT can also catalyse the conversion from 7-methylxanthine to theobromine by 3-N methylation (Mizuno et al., 2003b). XMT, MXMT and DXMT all use S-adenosyl-L-methionine acts as the methyl group donor, leaving *S*-adenosyl homocysteine as a by-product (Mizuno et al., 2003a).

Paraxanthine (1,7-methylxanthine) was proposed as an alternative immediate precursor to caffeine, similar to theobromine (Ashihara et al., 1996a). Catalysation of the conversion from paraxanthine to caffeine (step VI, Fig. 1) was shown in vitro by MXMT with low activity (Uefuji et al., 2003). However, MXMT cannot methylate the 1-N position of 7-methylxanthine and requires an additional enzyme to convert 7-methylxanthine into paraxanthine (step V, Fig. 1) (Ogawa et al., 2001). Results from Uefuji et al. (2003) raised questions about whether paraxanthine synthase (step V, Fig. 1) is present in the coffee genome, suggesting paraxanthine may not be synthesized in planta and cannot act as a precursor to caffeine. However, DXMT was speculated to act as paraxanthine synthase, converting 7-methylxanthine into paraxanthine by 1-N methylation (Yue and Guo, 2014). The affinity of DXMT towards the methylation of the 3-N position of 7-methylxanthine is higher than the methylation of the 1-N position. Therefore, 7-methylxanthine is primarily converted into theobromine rather than paraxanthine. Additionally, the 3-N methylation activity of DXMT was shown to also function in the conversion of paraxanthine to caffeine (Uefuji et al., 2003). The high affinity of DXMT to convert paraxanthine into caffeine could prevent any possible paraxanthine accumulation by its immediate conversion into caffeine (Yue and Guo, 2014).

#### Enzymes involved in the caffeine pathway in *C. Arabica* and *C. canephora*

As mentioned above, there has been no full agreement yet about the caffeine pathway. Moreover, several distinctions have been described between the coffee species *C. canephora* and *C. arabica*. In this chapter, the differences in the amino acid composition of the involved proteins as well as the transcription levels of the underlying genes are illustrated. This generates further insight into the alterations in functionality and specificity of the involved proteins. Due to the difference in genome composition and the number of chromosomes between *C. canephora and C. arabica*, certain genes in the pathway differ between the two species. *C. arabica,* being an allotetraploid species, contains two sets of homoeologous chromosomes originating from different species and consequently comprises two sets of methyltransferase genes.

**Fig. 2:**
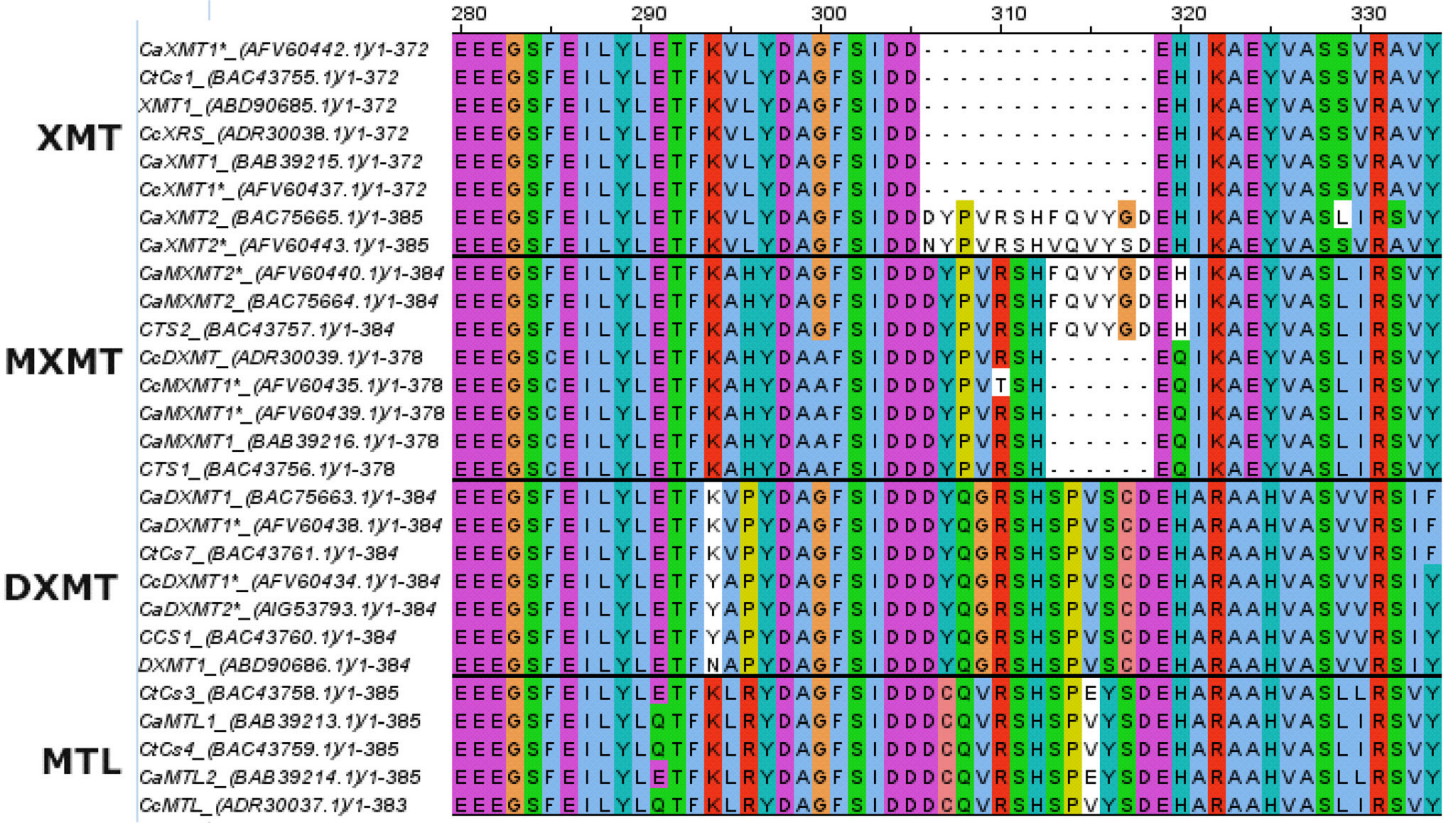
Multiple sequence alignment of partial methyltransferase protein sequences showing enzyme-specific deletions and substitutions. Overview of the deletion found at position 305 (Based on CaXMT1*) in methyltransferase proteins. Protein names are followed by accession codes (GenBank), amino acids are coloured according to ClustalX. Protein sequences are clustered according to sequence alignment and function; XMT = Xanthosine 7-methyltransferase; MXMT = 7-methylxanthinemethyltransferase; MTL = Methyltransferaseslike; DXMT = 3,7-dimethylxanthine methyltransferase

Perrois et al. (2014) identified three N-methyltransferases in *C. canephora* and six N-methyltransferases in *C. arabica* (Appendix 2). The amino acid sequence of these and previously described proteins in both *C. arabica* (Ca) and *C. canephora* (Cc) was compared in this review in an alignment (Fig. 2). The alignment also included other *C. arabica* proteins related to caffeine biosynthesis: CaXMT1, CaMXMT1, CaDXMT1, CaMXMT2 and CaXMT2 from Uefuji et al. (2003); methyltransferase-like (MTL) proteins CaMTL1 and CaMTL2 from Ogawa et al. (2001); and CtCs7 (*Coffea* tentative Caffeine synthase), CCS1 (*Coffea* caffeine synthase), CTS1 (*Coffea* theobromine synthase) and CTS2 from Mizuno et al. (2010). Furthermore, XMT1 and DXMT1 from McCarthy et al. (2007) were included for *C. canephora*. In addition, the alignment in Appendix 3 included the tentative proteins CtCS1, CtCS3, and CtCS4 found in *C. arabica* by Mizuno et al. (2010) and CcXRS (7-methylxanthosine synthase), CcMXMT, CcMTL, and CcDXMT found in *C. canephora* by Mohanan et al. (2014). Based on the Multiple Sequence Alignment (MSA) (Fig. 2, Appendix 3) and the described function of the proteins, the proteins were divided into four clusters: XMT (cluster I), MXMT (II), Methyltransferase-like (MTL) (III), and DXMT (IV). All previously mentioned proteins were clustered according to their relative amino acid sequence similarity (Appendices 3, 4).

Amino acid sequence identity between all proteins was high (> 79%), with 93–100% sequence identity within the XMT, MXMT, other MT and DXMT clusters (McCarthy et al., 2007; Mizuno et al., 2010; Ogawa et al., 2001; Uefuji et al., 2003). The sequence similarity of the proteins CaXMT1, CaXRS1 and CcXRS was 100%, as was that of CaMXT1, CaMXT1* and CcMXT and between CaDXMT1 and CADXMT1*. This indicates that these are the same protein.

CaXMT1, CaMXMT1 and CaDXMT1 were proven to form homodimers and heterodimers in vivo (Kodama et al., 2008; McCarthy et al., 2007). The ability to form heterodimers may be caused by the high sequence similarity between the proteins, although there are still significant differences within clusters. All proteins in the XMT cluster have a deletion of 13 amino acids after position 305 when compared to the other methyltransferases involved in the caffeine pathway; however, CaXMT2* and CaXMT2 have no deletion but a sequence highly similar to CaMXMT2*, CaMXMT2 and CTS2. These MXMT proteins also distinguish themselves from other proteins in the MXMT cluster because they lack the deletion of six amino acids after position 312 (Fig. 2). CaXMT2, CaXMT2*, CaMXMT2*, CaMXMT2 and CTS2 are therefore proposed to have a common ancestry within the *C. eugenioides* (Perrois et al., 2014). This is further illustrated by the high similarity between CcXMT1*, CaXMT1*, CcXRS and CaXMT1 and a high similarity between CcMXMT1, CaMXMT1*, CaMXMT1 and CTS1, indicating these originate from *C. canephora*. In the DXMT (III) cluster, the two sub-genomes are also found, with CcDXMT1*, CcDXMT, CCS1 and CaDXMT2* clustering, coming from the *C. canephora* genome and CtCs7 with CaDXMT1 and CaDXMT1*, coming from the *C. eugenioides* genome.

#### Active sites of enzymes involved

The enzymes in the DXMT cluster have both 1-N and 3-N methylation activity (McCarthy et al., 2007; Mizuno et al., 2010). The 3-N methylation activity of DXMT (or in CCS1) was found to be associated with a histidine on position 160 (position 161 in CaXMT1*) (Mizuno et al., 2010). Later, Yue and Guo (2014) suggested that this position allows for the acceptance of a proton from the hydroxy group of both 3-N methylation of 7-methylxanthin and the 1-N methylation of theobromine. The His160 is also found in CaXMT2* and all the proteins in the MXMT cluster, indicating it likely functions similarly in the methylation activity of those proteins. However, Mizuno et al. (2010) proposed that the difference in H160’s neighbouring amino acids causes the lack of 1-N methylation by MXMT. In the same way, the mutation of residue H219 in DXMT sequences will suppress its 3-N methylation activity. The 13 amino acid deletion found in the XMT proteins may cause 7-N methylation activity, but CCS1 mutants with the same deletion did not display catalytic activity towards 7-methylxantine (Mizuno et al., 2010).

#### Expression of genes involved throughout coffee plants

The catalytic activity of the N-methylation enzymes and the biosynthesis of caffeine and its precursors depend on the transcription level of the three *N*-methyltransferase genes. In *C. arabica* different expression patterns were found for homoeologous genes of *C. canephora* and *C. eugenioides* ancestry*.* Similar differences in gene expression of homoeologous genes were observed between *C. canephora* and *C. arabica*. This variation in gene expression contributes to the variation in caffeine concentrations in the harvested beans of different species and varieties. Caffeine biosynthesis activity during leaf development is lower than during the maturation of the beans (Ashihara et al., 1996a). In the early stages of leaf development in *C. canephora*, the levels of *CcXMT*, *CcMXMT* and *CcDXMT* are significantly elevated compared to their levels in mature leaves (Perrois et al., 2014). Overall expression of *CcXMT*, *CcMXMT* and *CcDXMT* in *C. canephora* coffee beans decreases as the beans mature, with *CcMXMT* having a low expression at all stages of development. In *C. arabica,* the *C. eugenioides* genes had significantly lower expression levels than their *C. canephora* counterparts, which in turn all had lower expression levels than in *C. canephora* plants (except *CaXMT1*, which was more expressed in the leaves of *C. arabica*). This indicates that in *C. arabica*, the genes derived from the *C. canephora* genome are under a different transcriptional regulation as those from the *C. eugenioides* genome.

#### Knocking out *XMT*

The first step in the main caffeine biosynthesis pathway is the conversion from xanthosine to 7-methylxanthosine by XMT (step I, Fig. 1) and could be a viable target for knock-out. No other enzymes were reported to be involved in this conversion. XMT’s additional functionality of converting xanthosine to 7-methylxanthine directly was described, nullifying the need for xanthosine nucleosidase (McCarthy and McCarthy, 2007). However, no other studies have since confirmed or reported on the xanthosine nucleosidase activity of XMT.

The knock-out of the *XMT* gene in coffee plants could be a valid strategy to knock-out the entire caffeine biosynthesis. No involvement of XMT in other pathways has been described and XMT only catalyses the initial step from the purine metabolism to the caffeine biosynthesis pathway (Mizuno et al., 2003a). This makes *XMT* an interesting target for knocking out the caffeine biosynthesis. Without XMT, the main pathway for caffeine cannot be initiated. However, a minor pathway through 3-methylxanthine (Fig. 1) may by-pass the requirement for XMT and allow for theobromine and subsequently caffeine to be produced (Ashihara and Crozier, 1999). The presence of 3-methylxanthine methyltransferase or enzymes with such activity has not been studied, so the occurrence of the step from xanthine to 3-methylxanthine is unknown *in planta*.

Crossing of *C. canephora* with the naturally caffeine-low *C. eugenioides* resulted in a hybrid that appeared deficient in *XMT* (Nagai et al., 2008). Xanthosine was reportedly converted into xanthine and taken up by the purine metabolism. As a result, no xanthosine accumulation was observed without caffeine being produced. The conversion from xanthosine into xanthine was also reported in two previously mentioned species from Madagascar: *C. millotii* and *C. perrieri* (Deng et al., 2017). The gene *XMT* is considered as a suitable candidate as target for knocking out the caffeine pathway.

#### Knocking out *DXMT*

The DXMT enzyme is a dual-functioning enzyme that can convert 7-methylxanthine into theobromine by 3-N methylation (step III, Fig. 1). The literature reviewed showed that DXMT is the only known enzyme to catalyse 1-N methylation of theobromine into caffeine (step IV, Fig. 1) in coffee plants. Knocking out the function of the DXMT enzyme will therefore disable the theobromine conversion into caffeine. In addition, the conversion of 7-methylxanthine into theobromine (given that MXMT is present) will be partially disabled. The 1-N methylation activity of DXMT towards 7-methylxanthine, as suggested in the model of Yue and Guo (2014), would also be disabled, completely blocking the synthesis of paraxanthine in *Coffea* plants (step V, Fig. 1). No evidence of alternative synthases for paraxanthine was found in *C. arabica* (Uefuji et al., 2003), further indicating that this pathway, if present, would not function in *DXMT* knock-out plants.

AC1, a natural caffeine-free variant of *C. arabica* described by Silvarolla et al. (2004), was found to have high levels of theobromine and low levels of caffeine when compared to the *C.* *arabica* cv. Mono Novo. The AC1 coffee plants contain an altered *DXMT* homologue ‘*CCS1*’, which has an altered substrate selection site, likely making it unable to bind to theobromine (Maluf et al., 2009). Reduced theobromine synthase activity was also measured in AC1 plants, with two times higher activity in ‘Mono Novo’ than in AC1 (Benatti et al., 2012). This is due to either the downregulation of transcription as suggested by Maluf et al. (2009) or the loss of function of *DXMT* (Benatti et al., 2012). In the latter case, all theobromine synthase activity is due to activity by *MXMT*.

With *DXMT* knocked out, theobromine would build up in the coffee plants as no catabolic pathway for theobromine is known in *C. arabica* and *C. canephora*. Evidence of a (low- activity) catabolic pathway for theobromine can be found in the related species *Coffea millotii*, although *C. millotii* does not synthesize theobromine or caffeine itself (Deng et al., 2017). As the caffeine biosynthesis pathway in *C. millotii* appears to halt at 7-methylxanthine*, *this could be considered as anecdotal evidence that functional *MXMT* and *DXMT* genes may not be present in *C. millotii*. Moreover, the underlying functionality and mechanisms of this potential pathway are unknown.

For blocking caffeine biosynthesis, *DXMT* is considered a potential candidate for producing knock-out mutants although theobromine may accumulate.

#### Knocking out *MXMT*

The MXMT enzyme converts 7-methylxanthine into theobromine via 3-N methylation (step III, Fig. 1), which can also be catalysed by DXMT. Knocking out *MXMT* will result in reduced theobromine formation in *C. arabica* and *C. canephora* but will not stop the theobromine formation entirely. DXMT can still facilitate the conversion from 7-methylxanthine into theobromine and subsequently into caffeine, maintaining the caffeine production in the plant. Downregulation of *MXMT* was performed using RNA interference (RNAi), resulting in a 70% reduction of caffeine in transgenic *C. canephora* plants and a nearly total reduction of caffeine in embryogenic tissues of *C. arabica *(Ogita et al., 2003, 2004). However, transcripts of both *XMT* and *DXMT* were also reduced in addition to *MXMT* transcripts, indicating that the entire pathway was repressed and not just *MXMT*. This is most likely due to the high sequence similarity between the genes, resulting in RNAi’s silencing of all three genes. As previously mentioned, *MXMT* has a very low expression in the beans of both *C. arabica* and *C. canephora* (Perrois et al., 2014). It is possible that DXMT takes over the 3-N methylation step of 7-methylxanthine (Step III, Fig. 1), effectively making DXMT responsible for the conversion of 7-methylxanthine to caffeine. Because of this, MXMT is considered a less interesting candidate for producing knock-out mutants for blocking caffeine biosynthesis in coffee.

#### Proposed guide RNAs

**Fig. 3:**
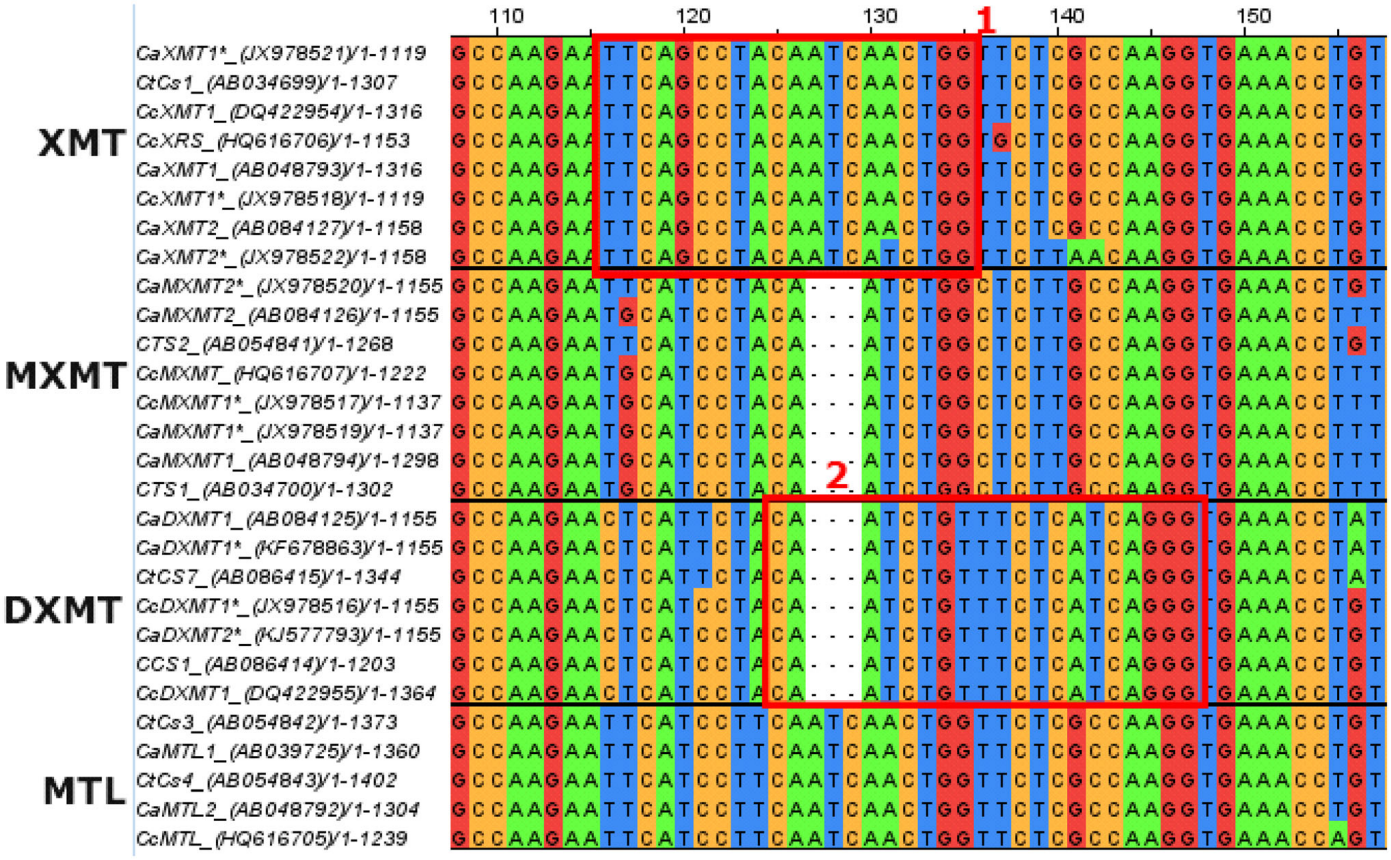
Multiple sequence alignment of methyltransferase genes with guide RNA target sites. Overview of the putative guide RNA target sites indicated by red boxes in the XMT and DXMT proteins. Region 1 is specific towards *XMT* and region 2 is selective for *DXMT*. Protein names are followed by accession codes (GenBank), nucleotides are coloured according to ClustalX. Genes are clustered according to sequence alignment and function; XMT = Xanthosine 7-methyltransferase; MXMT = 7-methylxanthine methyltransferase; MTL = Other methyltransferases; DXMT = 3,7-dimethylxanthine methyltransferase

To knock-out these candidate target genes, CRISPR-Cas9 can be used because it was previously proven to edit the genome of *C. canephora* succesfully (Breitler et al., 2018). For this, specific guide RNAs can be selected that target conserved regions of the methyltransferase genes, resulting in simultaneously targeting multiple methyltransferase family members. Alternatively, guide RNAs are employed to target a unique gene-specific sequence, thereby knocking out a single family member. In Fig. 3, two putative gene-specific regions for Cas9-targeting are highlighted in a section of the MSA. Due to the lack of the “ATC” (127–129) sequence in the clusters of *MXMT* and *DXMT*, region 1 is specific for *XMT*. On the other hand, region 2 is selective for *DXMT* because of small differences such as T131, C140, A141, T142 and G145 when compared to the *XMT* and *MXMT* gene clusters. Targeting these conserved nucleotide stretches specific for the respective gene cluster could lead to efficient and directed molecular modelling/mutagenesis with CRISPR-Cas9.

Other proposed regions are shown in Appendix 5. In addition to region 2, regions 3, 5 and 6 display characteristic variations in the arrangement of nucleotides, which makes them suited to focus on *DXMT* editing. Lastly, region 4 emphasizes a high sequence similarity in the three methyltransferase gene families found in the investigated *Coffea* species. The difference between the *XMT* and *DXMT* groups compared to the *MXMT* genes at position 480 (A versus G) could be used to target the first two clusters simultaneously. However, pursuing region 4 has a high likelihood of affecting all *MT* genes by CRISPR-Cas9 activity.

### Genetic modification of *C. arabica* and *C. canephora*

A genetic modification step is required to deliver the CRISPR-Cas machinery into the coffee plants. However, the limited genetic modification research done on coffee was mainly aimed at gene silencing by RNAi. The introduction of genes of interest was performed using particle bombardment or by *Agrobacterium tumefaciens*-mediated transformation methods. For coffee, only one gene editing experiment using CRISPR-Cas9 has been described, which was performed on the diploid *C. canephora*. No studies with CRISPR-Cas have been reported on the tetraploid *C. arabica*. Genetic differences between the two species may affect the modification success by CRISPR-Cas. Several articles mention that the modification of specific genes of interest, such as the knock-out of the *CcPDS* gene (Breitler et al., 2018), negatively affected the coffee plant’s viability. However, others mentioned no significant differences between the genetically modified coffee plants and the non-GM clone they produced, such as by introducing the green fluorescent protein (GFP) gene (Mishra et al., 2010).

#### Particle bombardment

One method that has been used since 2010 to introduce new genes into the plant genome is the biolistic delivery technique. This technique was utilised to introduce *Cry1Ac* from *Bacillus thuringiensis*, a gene resistant to the coffee leaf miner (*Leucoptera coffeella*) in *C. arabica* (De Guglielmo-Cróquer et al., 2010). *Cry1Ac* produces a crystalline protein which is highly toxic to the *L. coffeella* larvae. The whole plasmid carrying the *Cry1Ac* gene was transferred with a low-pressured helium pistol into somatic embryos of *C. arabica*. From 12 embryos that were transformed by De Guglielmo-Cróquer et al. (2010), only one surviving sample showed expression of the *Cry1Ac* gene.

Particle bombardment was also used by Barbosa et al. (2010) to transform *C. arabica*. Here, the α-amylase inhibitor-1 gene (*a-AI1*) from the common bean (*Phaseolus vulgaris*) was transformed into *C. arabica* calli. This gene infers resistance to the coffee berry borer (*Hypotheneumus hampei*). A seed-specific phytohemagglutinin promoter (*PHA-L*) was used in this study to ensure that the expression of the *a-AI1* gene was restricted to the seeds. The *a-AI1* gene was constructed as a DNA plasmid and attached to the surface of 1.2 μm microparticles. The microparticles were subsequently bombarded into the embryogenic callus of *C. arabica*. 26 plantlets were treated, 6 of which showed expression of the *a-AI1* gene. Among these 6 plants, 1 exhibited 88.86 ± 14.34% inhibition of *H.* *hampei* α-amylase due to the high expression of *a-AI1.* Nevertheless, after three years of greenhouse cultivation, sterility was detected in 50% of the transformed plants. Sterility might be caused by genetic variation within the cells (tissue culture effect) or the location of the *a-AI1* gene in the plant genome (position effect).

The latest publication using particle bombardment in coffee was conducted by Valencia-Lozano et al. (2019). The *Cry10Aa* gene, another resistance gene from *B. thuringiensis,* was bombarded into somatic embryos of *C. arabica*. In this experiment, several culture media were used with different sugars and concentrations (sucrose, mannitol and sorbitol) as the pre- and post-bombardment medium for the somatic embryos. Higher transformation efficiency was reported in this publication (up to 25.6%) with the treatment 6% of sucrose. The expression of Cry10Aa protein was proven to be stable until the last day of observation (three months). From the 17 putatively transformed plants, different relative expression of *Cry10Aa* among plants might have been due to multiple independent insertions.

#### *Agrobacterium tumefaciens*-mediated transformation

Nowadays, the *Agrobacterium tumefaciens* transformation method is the most frequently used technique to introduce a gene of interest into coffee plants. This method has the advantage of having a higher transformation efficiency and a more directed and stable integration of the gene of interest compared to methods like particle bombardment (Gelvin et al., 2009 as cited by Mohanan et al., 2014).

Several articles describe *A. tumefaciens* transformation experiments being performed on either *C. canephora* or *C. arabica*. In 2010, Sridevi et al. reported an *A. tumefaciens*-mediated transformation experiment performed on *C. canephora*. (Sridevi et al., 2010). In that same year Mishra et al. (2010) claimed the *A. tumefaciens* strain EHA105 to be more virulent on coffee than other *A. tumefaciens* strains. (Mishra et al. 2009 as cited by Mishra et al., 2010). Others noted that the *GFP* gene introduced into *C. canephora*, which was used as a visual marker, has cytotoxic effects (Haseloff and Siemering, 1998; Murray et al., 2004 as cited by Mishra et al., 2010), resulting in negative effects on the regeneration of transformed plants. However, in the study by Mishra et al. (2010) no negative effects from introducing the *GFP* gene were found on the plant regeneration; other researchers also found no negative effect (Pang et al., 1996; Leffel et al., 1997; Ghorbel et al., 1999; Tian et al., 1999; Molinier et al., 2000; Zhu et al., 2004 as cited by Mishra et al., 2010). Further *A. tumefaciens*-mediated transformation experiments on *C. canephora* mention both the successful introduction of the *Bacillus thuringiensis* c*ry1Ac* gene to create a variety resistant to the coffee leaf miner (*Leucoptera coffeella)* (Perthuis et al., 2015) and the introduction of promoter elements for the *CcSERK1* gene to investigate their role during somatic embryogenesis (Jiménez-Guillen et al., 2018; Pérez-Pascual et al., 2018). Perthuis et al. (2015) confirmed the statement of Mishra et al. (2010) that no growth difference was observed between the non-transformed clones and their transformed clones. Therefore, genetic modification itself does not seem to negatively affect the growth of the coffee plant (Perthuis et al., 2015).

Several *A. tumefaciens*-mediated transformation experiments were also performed on *C. arabica.* The technique was used to introduce both a stable gene resistant to the antibiotic hygromycin, used to select transgenic events (Ribas et al., 2011) and constructs containing the promotors for the *CaWRKY1a* and *CaWRKY1b* genes to investigate their role in (pathogen-related) stress responses (Petitot et al., 2013). Similarly, the technique was used to form transgenic plant lines to investigate the effects of three *CcDREB1D* promoter haplotypes that play a role in the expression of genes involved in drought-tolerance mechanisms in *C. arabica* (Alves et al., 2017). Only one article was found that discussed the *A. tumefaciens*-mediated transformation method for CRISPR-Cas9 gene editing. This experiment was performed on *C. canephora* (Breitler et al., 2018). No experiments with *A. tumefaciens*-mediated transformation for introducing CRISPR-Cas9 in *C. arabica* were found.

#### RNAi silencing

RNA interference (RNAi), also known as post-transcriptional gene silencing, is a mechanism to silence the expression of specific genes by degrading the targeted mRNA so no protein is produced. Mohanan et al. (2014) investigated RNAi on the N-methyltransferase (NMT) multigene family, genes that play a role in caffeine biosynthesis, in *C. canephora*. When embryos were transformed, caffeine content was reduced by 90%; however, this result did not last long. Seven-month-old and eight-month-old transformed plants showed only a 15–20% reduction in caffeine content. According to two articles (Furutani et al., 2007 and Kalantidis et al., 2002), the production of RNAi is influenced by plant development and environmental conditions that are still ambiguous. Mohanan et al. (2014) suggested that the effectiveness of RNAi can be increased by a tighter hairpin loop (90 bp between two arms of the invert repeat).

#### CRISPR-Cas9 gene editing

The proof-of-concept article by Breitler et al. (2018) discusses the use of the CRISPR-Cas9 gene editing technique in *C. canephora* to emphasize the benefits of the ‘Coffee gRNA Identification Program’ (CRIP) programme, by knocking out the phytoene desaturase gene (*CcPDS*). This gene is used as a ‘model gene’ with a full knock-out mutation resulting in in vitro albino plants due to its role in carotenoid and chlorophyll biosynthesis. Targeted mutagenesis was achieved by creating targeted double-strand breaks in the gene, resulting in putative frame shift mutations due to errors made by the cell repair mechanisms. A more specific gene-targeting approach using CRISPR-Cas9 is nowadays possible since the genome of *C. canephora* has been sequenced (Denoeud et al., 2014 as cited by Breitler et al., 2018). For the proof-of-concept experiment, embryonic cultures were transformed using the *A. tumefaciens* transformation method to introduce the CRISPR-Cas9 system into the plant to generate targeted mutations in the *CcPDS* gene. Successful knock-outs of the *CcPDS* gene were easily confirmed by the formation of an albino phenotype. A mutation efficiency of 30.4% was reported, split into 22.8% heterozygous and 7.6% homozygous mutants based on the sequence comparison of individual clones. The development of fully grown plants from the transformed embryonic cultures was drastically reduced with strong pigmentation changes, but full albino phenotypes were not observed. The cause of the developmental issues was indicated to be related to the choice of the gene of interest and not to the use of the CRISPR-Cas9 technique itself (Breitler et al., 2018). Although the experiment was not completely successful, it demonstrated the efficacy of the CRISPR-Cas9 technique combined with the *A. tumefaciens-*mediated transformation method and followed by the recovery of fully-grown plants from the modified embryonic cell cultures. Therefore, CRISPR-Cas9 could be a feasible method for modifying.

#### Non-transgenic CRISPR-Cas mutants

Following the design and preparation of the CRISPR-Cas9 machinery, the plasmid carrying the sequences for the gRNA(s) and Cas9 protein is introduced into the *Coffea* plant via *A. tumefaciens*-mediated transformation, as pointed out by Breitler et al. (2018). After the successful transformation of a gene, the gene modification step occurs and the effectively altered crops are then selected. However, the duration of the last step is determined by screening and breeding procedures. Thus, resistance genes such as antibiotics are used as selectable markers to hasten the process by clearly indicating the modified crops. Subsequently, the selected mutants require selfing or backcrossing to fully eliminate the occurrence of any marker genes or used guide RNAs of the gene editing tool introduced as part of the CRISPR-Cas9 machinery. This extra step ultimately prolongs breeding and displays a certain obstacle, since coffee is a vegetatively propagated highly heterozygous crop and backcrossing is not advisable.

The widely used transformations with *A. tumefaciens* inject so-called “T-DNAs” to invasively modify the plant, leaving a significant trace of the modification. In contrast, when applying a transient T-DNA expression, components of the CRISPR-Cas9 system such as gRNA, donor-DNA and Cas proteins need only to be expressed to induce genetic modifications and will degrade over time. Thus, the CRISPR-Cas9 system can provide targeted mutations while avoiding the integration of transgenes into the plant genome. With this in mind, non-transgenic mutant plant lines can be generated by employing CRISPR-Cas9.

Moreover, CRISPR-Cas9 illustrates a relatively high specificity and is only present in the first generation. As stated in Zhang et al. (2019), the thorough assessment of potential off-target sites led to the minimization of false editing. Proper gRNA design and whole genome screening resulted in no detectable mutations at the potential off-target sites. This was proven by sanger sequencing (Zhang et al. 2019). Since the gene editing takes place in the generation 1 plant, after reproduction, part of the offspring originating from the initially transformed plant does not contain any CRISPR-Cas9 gene or transgene traces anymore, except an altered genome at the CRISPR target site. In conventional crossbreeding and breeding approaches, crossing leads to the segregation of favorable gene combinations. However, these approaches are still coherently linked to very time-consuming practices and can potentially result in the loss of elite variety characteristics. As an alternative, CRISPR-Cas-induced gene editing provides more reliable, faster and more efficiently genetically altered crops without jeopardizing the plants or the end consumer’s health or integrity due to its reliability on-target cleavage/editing.

Compared to the use of T-DNAs, our proposed approach does not insert a genetic fragment into the genome of the plant but knocks-out a specific gene to interfere with caffeine production. As the non-integrated transgenes will be degraded over time, non-transgenic cells with mutations will remain; these cells can eventually be regenerated into non-transgenic mutant plants.

## Discussion

The main findings of the retrieved literature are presented below. First, observations made during the uncovering of the caffeine biosynthesis pathway are discussed and candidate target genes are further evaluated. Next, genetically modifying coffee plants is further examined. Finally, the expected taste of caffeine-free coffee as well as the social acceptance and legislation of genetically modified organisms are considered.

### Caffeine biosynthesis

As evident from the MSA (Fig. 2 and Appendix 3), multiple genes and enzymes are involved in the caffeine biosynthesis pathway (Fig. 1). Because different studies have used various names for identical enzymes or enzymes with high sequence similarity, uncovering and describing the full story is more complicated. This review aimed to shed light on both the current understanding of biosynthesis and how the pathway can be utilized or modified to create caffeine-free coffee plants.

Multiple studies have suggested that the expression of certain genes involved in caffeine biosynthesis may be tissue-specific. Genes that are primarily transcribed in the fruits (beans) of coffee plants could be excellent candidate target genes. For instance, Mizuno et al. (2003b) showed that *CCS1* is transcribed in both the leaves and fruits of *C. arabica,* whereas *CaDXMT is* primarily present in the fruits of *C. arabica* (Uefuji et al., 2003). However, the presence of *CaDXMT* in *C. arabica* leaves was shown in a later study (Maluf et al., 2009). Silencing these genes would only affect the caffein biosynthesis in coffee beans and ultimately the beverage produced by further manufacturing processes. Other plant tissues, such as leaves, would be (largely) unaffected by knocking out one of these genes and they would continue to produce caffeine to maintain a defence against pests. However, the transport of caffeine throughout the plant and into its fruits may also be a possible target, but this subject was not reviewed here. More research is required to identify whether caffeine transport is present and to confirm whether certain genes are truly unique to the coffee beans.

#### Minor caffeine biosynthesis pathways

Caffeine biosynthesis consists of more than the main pathway; multiple minor pathways leading to caffeine have been proposed (Fig. 1). The presence of a minor pathway through paraxanthine had already been suggested by Ashihara et al. back in 1996. However, there have been doubts about whether paraxanthine is truly involved in the biosynthesis of caffeine in planta (Uefuji et al., 2003). Paraxanthine was shown to be the preferred substrate for DXMT (Roberts and Wallert, 1979), yet theobromine is primarily converted *in planta* and no evidence of the *in planta* synthesis of paraxanthine has been reported since (Uefuji et al., 2003).

Ashihara and Crozier (1999) proposed a minor pathway for caffeine biosynthesis from xanthine to 3-methylxanthine, followed by 7-N methylation to yield theobromine (Fig. 1). Normally, 3-methylxanthine is part of the caffeine catabolism pathway, but here it was suggested that caffeine can also be produced through 3-methylxanthine. However, the enzyme responsible for the 7-N methylation of 3-methylxanthine was not mentioned. Additionally, the species of *Coffea* in which this pathway might be present were not described, although the full article was on natural caffeine-low species. This minor pathway could be similar to the paraxanthine minor pathway (steps V and VI, Fig. 1) where the main pathway is prioritized *in planta* and the activity of the minor pathway is rarely observed.

#### Multiple sequence alignment

The MSA showed highly conserved regions in the N-methyltransferases involved in the caffeine pathway and also indicated the potentially active site residue His160 in the sequence, as suggested by Yue and Guo (2014). This key catalytic site of the MXMT and DXMT cluster accepts the proton of both 7-methylxanthine and theobromine in DXMT and could be a viable target for gene editing with CRISPR-Cas9. This would not cause the whole enzyme to be knocked out but would rather inhibit its N-methylation activity regarding the caffeine pathway.

The MSA also highlighted the fact that two sets of genes can be found within the *C. arabica* genome, one with *C. canephora* ancestry and one with *C. eugenioides* origin; each set displays a different sequence. This makes it possible to knock-out only one of the two genes present in *C. arabica*. As *C. canephora* genes are more expressed than those of *C. eugenioides* in both beans and leaves, knocking out only these genes might result in a variant with less caffeine. In allopolyploid wheat, it has already been shown that all copies of a gene can be knocked out, indicating that the alloploidy of *C. arabica* does not prevent it from being modified on a molecular level (Wang et al., 2019).

The MSA also showed high sequence similarity between both N-methyltransferases in the pathway and other MTLs not included in the pathway. The high similarity between N-methyltransferase was illustrated by the RNAi study by Ogita et al. (2004), which showed that silencing *MXMT* affected *XMT* and *DXMT* as well. Targeting sequences that are unique to the specific N-methyltransferase variant with high specificity is therefore needed to knock-out target genes and prevent off-targets in methyltransferases involved in other pathways.

#### Candidate gene target evaluation

A review of the literature identified three primary candidate genes as gene editing targets to block caffein biosynthesis in coffee: *XMT*, *MXMT* and *DXMT*. *XMT* could be a valid target gene for knocking out the caffeine biosynthesis pathway since XMT catalyses the first conversion in the pathway. Enzymes involved in subsequent steps would not have any substrate to act on. Additionally, no alternative enzymes for the conversion from xanthosine to 7-methylxanthosine were proposed, preventing the synthesis of 7-methylxanthine, theobromine and caffeine entirely. Alternative (minor) pathways through, for instance, 3-methylxanthine could still cause caffeine to be produced. Nevertheless, the existence of these minor pathways in either *C. arabica* or *C. canephora* has not been fully studied yet and further research is required. Minor pathways aside, removing the ability to synthesize XMT from coffee plants may result in xanthosine accumulation. Xanthosine degradation by conversion to xanthine was observed in *C. millotii* and *C. perrieri* from Madagascar (Deng et al., 2017). A similar pathway may be present in *C. arabica* and *C. canephora* but requires further investigation.

In contrast to *XMT*, *MXMT* is not likely to be a valid gene target for knocking out the caffeine biosynthesis in coffee plants. The function of MXMT was also shown as a secondary function of DXMT. Therefore, knocking out *MXMT* would not prevent the synthesis of theobromine and ultimately caffeine. In nature, no low-caffeine coffee plants lacking *MXMT* functionality have been described thus far, reinforcing the statement that *MXMT* is not likely to be a valid gene target.

Finally, *DXMT* can be a valid gene target. DXMT is involved in multiple steps in the caffeine biosynthesis pathway. Knocking out *DXMT* would prevent conversion from theobromine to caffeine (step IV, Fig. 1) entirely and partially disable the conversion from 7-methylxanthine to theobromine (step III, Fig. 1). Considering MXMT to remain functional, theobromine would still be formed and likely accumulate *in planta*. Evidence for the existence of (low activity) theobromine degrading enzymes was observed in *C. millotii,* even though *C. millotii* does not produce theobromine (Deng et al., 2017). Further investigation is required to determine whether such theobromine degradation enzymes are present in *C. arabica* and *C. canephora* and whether these enzymes can prevent theobromine accumulation.

Based on the MSA, several CRISPR-Cas guide RNAs were proposed to knock-out *XMT*, *DXMT* or both at once (Fig. 3, Appendix 5). The suggested guide RNAs target sites were not checked against the whole genome of *C. arabica* and *C. canephora* to assess whether off-target activity could occur. Other MTLs share the highest similarity with XMT and DXMT (as was evident from the MSA, Appendix 3). Therefore, off-target activity should be carefully checked for other MTL genes. Targeting a specific unique region such as region 2 (Fig. 3, Appendix 5) can avoid off-target activity on *MTL* genes by creating guide RNAs that span a gap in the *DXMT* sequence (compared to the MTL sequence). The overall complexity of the caffeine biosynthesis as well as the role, properties and interconnections of the N-methyltransferases can be studied by gene-specific targeting with CRISPR-Cas9. Our proposed target regions and ultimately CRISPR-Cas9 knock-outs may help to increase the understanding of the role, function and properties of key genes and proteins in the caffeine biosynthesis pathway.

#### Possible effects on the taste of coffee

As stated before, knocking out genes involved in the caffeine biosynthesis pathways might result in the accumulation of certain precursors, which can influence the taste of the final product. The knock-out of *DXMT* may lead to the accumulation of theobromine. Similar to caffeine, theobromine is a bitter-tasting alkaloid that is also present in cacao (Izawa et al., 2010) and so may compensate for the loss of caffeine-related bitterness in caffeine-free coffee. No studies were found that evaluated the difference in bitterness between caffeine and theobromine; however, a study by Baggott et al. (2013) compared the psychopharmacology affect (mood, cognitive performance and some physiological measures) of caffeine and theobromine. Baggott et al. (2013) showed that theobromine has a weaker effect than caffeine on alertness. Theobromine has clear health benefits; it widens blood vessels (thus decreasing blood pressure), has diuretic properties and stimulates heart activity (Izawa et al., 2010).

Knocking out *XMT* could result in an increase of xanthosine. No scientific reports discuss the effect of xanthosine on taste. An organoleptic test would be required to assess the taste profile of edited coffee which lacks *XMT* activity.

#### Alternative approach to produce caffeine-free coffee plants

An alternative approach to potentially produce caffeine-free (or caffeine-low) coffee plants would be to introduce a high-activity catabolism of caffeine. Ashihara and Crozier (1999) suggested that 7-N demethylase from *C. eugenioides* could be introduced into *C. arabica*. The catabolism of caffeine in *C. arabica* was shown to be blocked between caffeine and theophylline (1,3-dimethylxanthine), whereas the degradation of theophylline remains functional (Ashihara et al., 1996b). The introduction of 7-N demethylase would remove this blockade and allow the caffeine catabolism to decrease the caffeine content. The degree to which this would reduce caffeine contents is unknown, but the naturally caffeine-low species *C. eugenioides* is primarily caffeine-low through this mechanism. However, it should be mentioned that introducing an entire gene, albeit from another coffee plant, counts as a transgenic (or cisgenic) modification rather than gene editing and thus entails different legislation, acceptance and labelling of the end-product.

More caffeine-low species exist than have been mentioned/described in this review. For instance, Ashihara and Crozier (1999) described two more caffeine-low species in addition to *C. eugenioides*. For these caffeine-low species there is yet no clear evidence of which genes or enzymes were affected and/or missing. Further investigation is required to deduce how these plants maintain their low caffeine content and if this mechanism can be mimicked in *C. arabica* and *C. canephora*.

### Genetic modification of *C. arabica * and *C. canephora*

Different methods to introduce foreign DNA, like CRISPR-Cas9 plasmids, into cells of both *C. canephora* and *C. arabica* have been described.

Particle bombardment was used in the early 2010s to transform plants and has been employed to successfully transform *C. arabica.* However, several drawbacks of particle bombardments make this technique unreliable. Stress responses were triggered by the invasion of the microparticles into the plant tissue. This may explain the relatively low transgene expression after transformation (Lacroix and Citovsky, 2020). Particle bombardment results in a more random integration of the gene of interest into the plant cell, the gene might be introduced in a highly methylated part or a more condensed part of the chromosome, such as heterochromatin and the gene of interest might not always be expressed. Also, multiple copies of the transgene have often been reported to have been integrated into the plant genome, which will increase the chance of the silencing phenomenon by the plant itself and de novo methylation (Kohli et al., 2003).

The *A. tumefaciens*-mediated transformation method has largely replaced the particle bombardment method since it is much more reliable and efficient. This method of introducing the CRISPR-Cas9 system into plant cells is nowadays the most frequently used technique.

#### RNAi-mediated gene silencing in *C. canephora*

The RNAi silencing technique was used on *C. canephora* to target the N-methyltransferase (NMT) multigene family, which includes *XMT*, *MXMT* and *DXMT*. The partial reduction of the targeted gene expression resulted in a 20% increased of theobromine content while the caffeine content was lowered by 70–95% (Mohanan et al., 2014). Silencing of one gene in the caffeine pathway was proven difficult because of the high similarities between N-methyltransferase genes, causing RNAi to silence multiple genes (Ogita et al., 2003, 2004). This may possibly mean that RNAi silencing could have effects on N-methyltransferases outside of the caffeine pathway, potentially interfering with other biological functions of the plant. To produce a completely caffeine-free coffee variety, the RNAi technique is not the preferred method and molecular modelling tools with a higher target specificity are required.

#### CRISPR-Cas as a gene editing tool for *C. arabica* and *C. canephora*

Proof of concept for CRISPR-Cas9 gene editing on *C. canephora* using *PDS* as a model gene resulted in homozygous mutated plants (Breitler et al., 2018). However, due to the chosen gene of interest, the viability of the produced mutants was poor and the predicted phenotype of complete homozygous silencing was not observed. Using a similar strategy to modify the *C. arabica* genome may present more challenges due to its tetraploidy, requiring multiple allelic sequences to be modified for a complete knock-out mutation. No proof of concept for CRISPR-Cas gene editing on C. arabica was discovered during this review. Multiallelic gene editing has often been reported for other polyploid species. For example, experiments performed on tetraploid potato (*Solanum tuberosum* L.) cv. Desiree (Tuncel et al., 2019), hexaploid wheat (*Triticum aestivum*) cv. Fielder (Jouanin et al., 2020) and tetraploid Oilseed Rape (*Brassica napus*) (Braatz et al., 2017) underline the feasibility of effective CRISPR-Cas9 application in polyploid plant species. However, several studies such as Wolabu et al. (2020), Liu et al. (2018) and Ryder et al. (2017) pointed out the limits of multi-allelic targeting of genes in polyploid crops with the molecular modelling tool. These articles concur that completely knocking out a gene of interest in polyploid plants using CRISPR-Cas9 is possible, yet not guaranteed.

Successful mutagenesis of a gene of interest may also depend on the mode of action of the gene of interest itself (Breitler et al., 2018; Perthuis et al., 2015). If the gene plays a crucial role in the metabolism or the development into fully grown plants, gene editing may drastically affect the viability of the plant. However, since naturally caffeine-free coffee species are found in nature, caffeine metabolism is not likely to be crucial to the viability of coffee plants (Hamon et al., 2015). Therefore, in theory, complete knock-outs of genes involved in the caffeine pathway using CRISPR-Cas9 should be possible for both *C. canephora* and *C. arabica*.

Following the idea of gene editing in polyploid plants, Yasumoto et al. (2020) obtained mutant potato plants without integrating the TALENs transgenes by Agrobacterium-mediated transient expression in plant tissue and by omitting selection. For wheat, a similar method has been reported, following transient expression of DNA or RNA coding for the CRISPR-Cas9 genes in calli (Zhang et al., 2016). Ma et al. (2020) developed an interesting approach for genome editing without the need for transgene integration by making use of the viral delivery of CRISPR-Cas9. They engineered the Sonchus yellow net rhabdovirus (SYNV) for in planta delivery of CRISPR-Cas9 reagents and demonstrated mutagenesis and chromosome deletions at high frequency in infected allotetraploid tobacco. Using tissue culture technology, heritable, virus-free shoots could be regenerated from infected somatic tissue.

### Social hurdles

Up to this point, this systematic review has discussed the technical feasibility of producing gene- edited caffeine-free coffee beans. However, several factors, like social acceptance and legislation, need to be taken into account before gene edited coffee can become a commercial success (Borrell, 2012; Braun and Dabrock, 2018). Both legislation and social acceptance will now be discussed.

#### Legislation

The two ways of responding to gene-edited products involve adopting either process-based regulations or product-based regulations. The European market implies process-based regulation, while the US market illustrates the framework for product-based regulation (Ishii and Araki, 2017). The process-based regulations focus on the gene editing techniques used to alter a product. In 2018 the European Court decided that CRISPR-Cas gene editing falls under the current and strict EU rules for genetic modification. These rules point out that extensive testing must be carried out to determine whether a crop made with the help of CRISPR-Cas is safe for humans, animals and the environment. Such a crop must also be labeled as 'genetically modified food', so that consumers know what they are buying. Politicized decision-making has caused long delays in the authorization process for GM crops (Lucht, 2015). As a consequence, new legislation entails an intensive, time-consuming process (European Commission, 2021; European Parliament, 2016; Ishii, 2018). Therefore, selling gene-edited coffee in Europe will be challenging. On the other hand, product-based regulation does allow gene-edited food crops, as long as the product is transgene-free. Product-based GMO regulations state that all human and animal food from plants, even when genetic engineering is involved, are held to the same standards as all other foods produced, stored, shipped or processed in the USA (U.S. Food & Drug Administration, 2020).

#### Social acceptance

The second issue to consider is the incorporation of genetically engineered products into society. Since their discovery of the CRISPR-Cas system, Charpentier and Doudna have developed recommendations in consultation with several highly renown researchers on how to incorporate gene editing into society and increase transparency for consumers (Doudna and Charpentier, 2014). A few steps should be taken before decisions can be made in a new era in biology and genetics (Baltimore et al., 2015). This paper mainly describes the steps that should be taken into account when applying gene editing on humans. However, these steps are also partially applicable for closer goals, such as gene-edited crops. First, standard safety methods should be implemented to measure the efficiency and off-target effects of gene editing. Second, scientific experts and bioethics communities should inform and educate societies as a whole in a more accessible manner; for example, fora or other media could be used as long as the risks and rewards of such a powerful technology are explained with scientific, social, ethical and legal implications. Ultimately, this would lead to an increased knowledge about the properties, possibilities and risks of said molecular modelling techniques in our communities. Third, guidelines in gene editing should be made clear. This requires international policy makers and scientists to cooperate in developing what is (and what is not) ethically acceptable research. Fourth, appropriate oversight should be applied to laboratory work in terms of regulations to evaluate the efficacy of genome editing technologies. Hence, CRISPR-Cas can be used responsibly without inhibiting research and development. Finally, all of the purposes of gene editing techniques must be cautiously evaluated because of the unknown consequences. These crucial points should be applied to the discussed (putative) coffee crop, which means, among other things, that to create a commercially successful gene-edited caffeine-free coffee, independent experts should be consulted or worked with to ensure successful project execution and adequate outreach to guarantee consumer satisfaction.

#### Closing statement

The most feasible approach to create caffeine-free coffee species would be to knock-out genes in the caffeine biosynthesis pathway through CRISPR-Cas9, introduced by *A. tumefaciens*-mediated transformation. Producing caffeine-free *C. arabica* may prove harder than in *C.* *canephora* because of *C. arabica`s* tetraploidy. Having multiple copies of the target genes, but successful simultaneous targeting of multiple gene copies has been demonstrated in several other plant species already.

Three candidate target genes were evaluated while two (*XMT* and *DXMT*) were identified as potential candidates to produce caffeine-free coffee through gene editing. The conservation of gene sequences may enable targeting multiple N-methyltransferases simultaneously, although specific regions for targeting N-methyltransferases have been identified as well. Guide RNAs target sites to knock-out *XMT*, *DXMT* or both simultaneously were proposed. The possibility of precursor accumulation was evaluated and will likely not be a major obstacle in producing caffeine-free coffee plants. Xanthosine (if *XMT* is knocked out) can be degraded to xanthine, whereas theobromine (if *DXMT* is knocked out) may present putative health benefits. The third candidate gene, *MXMT*, was not identified as a valid gene target as the role of MXMT can also be fulfilled by DXMT due to its dual functionality. An alternative approach without CRISPR-Cas9 was then proposed by introducing the 7-N demethylase from *C. eugenioides* by A. tumefaciens-mediated transformation. However, this alternative emphasises introducing genetic information as an insert into the plant´s genome, whereas our proposed application simply generates a non-functional gene through knock-out. Instead of the extensive use of chemicals for decaffeination, the combination of CRISPR-Cas and *A. tumefaciens*-mediated transformation requires fewer resources and thus significantly improves sustainability, logistical organization, processing time and required materials.

## Appendices

Any lists or images mentioned in the text but not included immediately can be found here.

### Appendix 1: Final selection of retrieved literature

This final selection of the retrieved literature has been separated into articles used in ‘Genetic modification of *C. arabica* and *C. canephora*’ and ‘Modifying the caffeine biosynthesis pathway’. Only the author(s) and the year are mentioned here, similar to in-text references. For the full reference, please refer back to “References”.

**Table:** 

*State of the art literature shortlist*
Alves et al. (2017)
Barbosa et al. (2010)
Breitler et al. (2018)
Gamboa-Becerra et al., 2019
Guglielmo-Cróquer et al. (2010)
Jiménez-Guillen et al. (2018)
Kumar et al. (2018)
Mishra et al. (2010)
Mohanan et al. (2014)
Pérez-Pascual et al. (2018)
Perthuis et al. (2015)
Petitot et al. (2013)
Ribas et al. (2011)
Sridevi et al. (2010)
Valancia-Lozano et al. (2019)
Vargas-Guevara et al., 2018
*Caffeine biosynthesis pathway literature shortlist*
Ashihara and Crozier (1999)
Ashihara et al. (1996a)
Benatti et al. (2012)
Deng et al. (2017)
Kodama et al. (2008
Maluf et al. (2009)
McCarthy and McCarthy (2007)
McCarthy et al. (2007)
Mizuno et al. (2003a)
Mizuno et al. (2003b)
Mizuno et al. (2010)
Mohanan et al. (2014)
Mohanan et al. (2014)
Nagai et al. (2008)
Ogawa et al. (2001)
Ogita et al. (2003)
Ogita et al. (2004)
Silvarolla et al. (2004)
Uefuji et al. (2003)
Yue and Guo 2014)

## Appendix 2: Overview gene and protein sequences

**Table:** 

Name gene	mRNA sequence	Protein sequence	Author
*CaXMT1*	AB048793	BAB39215	Uefuji et al. (2003)
*CaMXMT1*	AB048794	BAB39216	Uefuji et al. (2003)
*CaDXMT1*	AB084125	BAC75663	Uefuji et al. (2003)
*CaMXMT2*	AB084126	BAC75664	Uefuji et al. (2003)
*CaXMT2*	AB084127	BAC75665	Uefuji et al. (2003)
CcDXMT1*	JX978516	AFV60434	Perrois et al. (2014)
CcMXMT1*	JX978517	AFV60435	Perrois et al. (2014)
CcXMT1*	JX978518	AFV60437	Perrois et al. (2014)
CaDXMT1*	KF678863	AFV60438	Perrois et al. (2014)
CaMXMT1*	JX978519	AFV60439	Perrois et al. (2014)
CaMXMT2*	JX978520	AFV60440	Perrois et al. (2014)
CaXMT1*	JX978521	AFV60442	Perrois et al. (2014)
CaXMT2*	JX978522	AFV60443	Perrois et al. (2014)
CaDXMT2*	KJ577793	AIG53793	Perrois et al. (2014)
*CaMTL1*	AB039725	BAB39213	Ogawa et al. (2001)
*CaMTL2*	AB048792	BAB39214	Ogawa et al. (2001)
*CcMTL*	HQ616705	ADR30037	Mohanan et al. (2013)
*CcXRS*	HQ616706	ADR30038	Mohanan et al. (2013)
*CcMXMT*	HQ616707	ADR30039	Mohanan et al. (2013)
*CtCs1*	AB034699	BAC43755	Mizuno et al. (2010)
*CTS1*	AB034700	BAC43756	Mizuno et al. (2010)
*CTS2*	AB054841	BAC43757	Mizuno et al. (2010)
*CtCs3*	AB054842	BAC43758	Mizuno et al. (2010)
*CtCs4*	AB054843	BAC43759	Mizuno et al. (2010)
*CCS1*	AB086414	BAC43760	Mizuno et al. (2010)
*CtCs7*	AB086415	BAC43761	Mizuno et al. (2010)
*CcXMT1*	DQ422954	ABD90685	McCarthy et al. (2007)
*CcDXMT1*	DQ422955	ABD90686	McCarthy et al. (2007)

### Appendix 3: Multiple sequence alignment


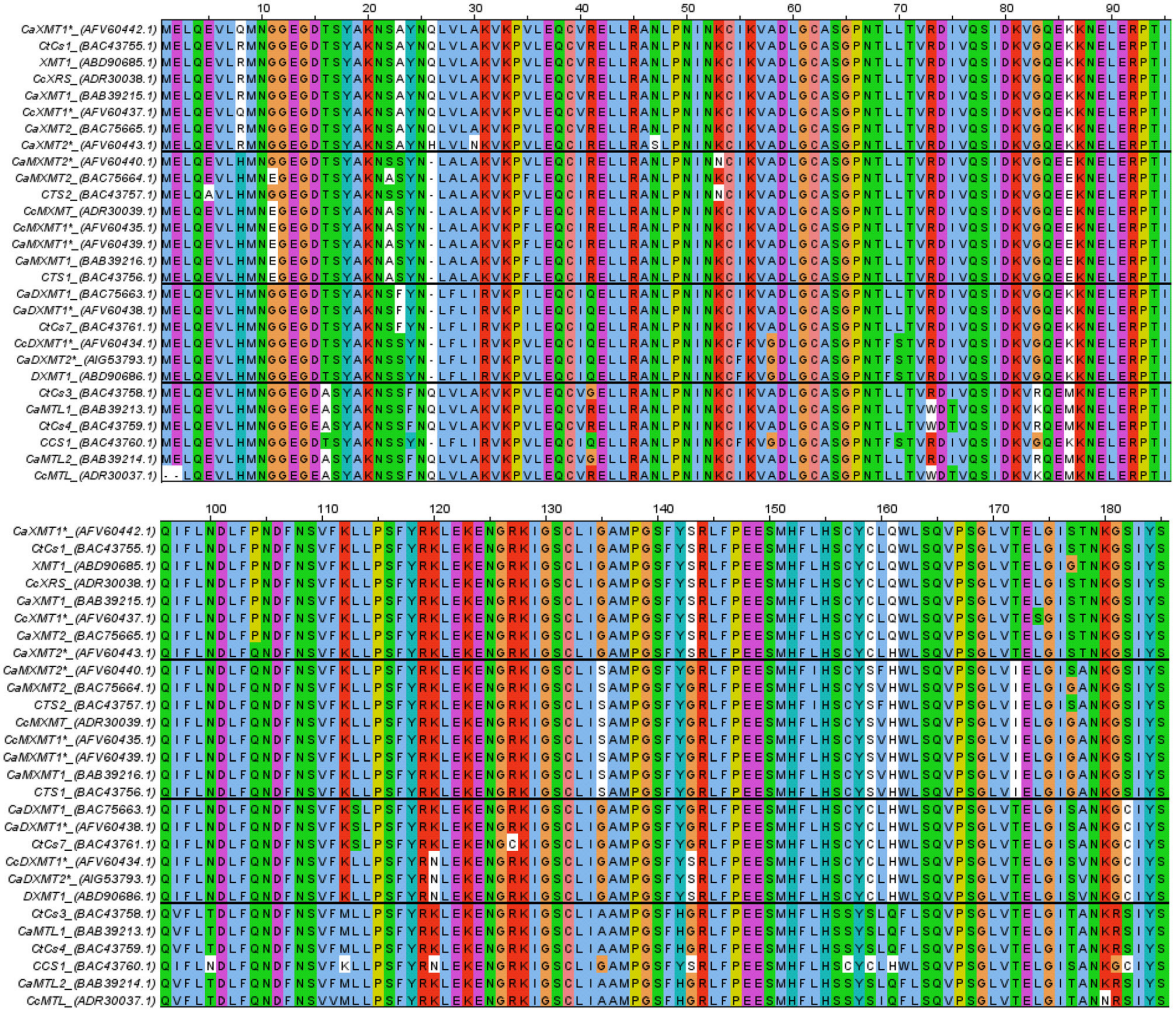


### Appendix 4: Amino acid positions supporting clusters

**Table:** 

Position^a^	Cluster XMT (I)	Cluster MXMT (II)	Cluster other MTL (III)	Cluster DXMT (IV)
16	T	T	A	T
24	Y	Y	F	Y
28	V	A	V	F
31	K	K	K	R
86	K	E	M	K
97	I	I	V	I
100	N	N	T	N
112	K	K	M	K
135	G	S	A	G
142	Y	Y	H	Y
157	C	C	S	C
159	C	S	S	C
162	W	W	F	W
172	T	I	T	T
181	G	G	R	G
182	S	S	S	C
191	L	P	P	P
193	V	V	V	I
212	H	H	R	H
214	E	K	E	E
217	F	F	L	I
219	H	R	R	R
226	C	C	C	F/W
230	G	V	G	E
235	A	E	G	H/N
238	A	P	T	S
243	E	D	E	E
245	A	A	A	S
267	V	F	I	I
268	Y	F	Y	Y
270	P	P	A	P
272	A	A	V	T
296	L	H	R	P
321	I	I	A	A
322	K	K	R	R
324	E	E	A	A
325	Y	Y	H	H
333	V	V	V	I
351	F	F	F	S
356	K	K	T	K
357	H	H	N	N
362	L	L	I	L
370	N	N	N	D
371	N	N	N	S

^a^Based on the CaXMT* sequence

### Appendix 5: Possible guide RNA target sites